# *Drosophila melanogaster* rhodopsin Rh7 is a UV-to-visible light sensor with an extraordinarily broad absorption spectrum

**DOI:** 10.1038/s41598-017-07461-9

**Published:** 2017-08-04

**Authors:** Kazumi Sakai, Kei Tsutsui, Takahiro Yamashita, Naoyuki Iwabe, Keisuke Takahashi, Akimori Wada, Yoshinori Shichida

**Affiliations:** 1 Department of Biophysics, Graduate School of Science, Kyoto University, Kyoto, 606-8502 Japan; 2 Department of Organic Chemistry for Life Science, Kobe Pharmaceutical University, Kobe, 658-8558 Japan; 3 Research Organization for Science and Technology, Ritsumeikan University, Kusatsu, Shiga 525-8577 Japan

## Abstract

The genome of *Drosophila melanogaster* contains seven rhodopsin genes. Rh1-6 proteins are known to have respective absorption spectra and function as visual pigments in ocelli and compound eyes. In contrast, Rh7 protein was recently revealed to function as a circadian photoreceptor in the brain. However, its molecular properties have not been characterized yet. Here we successfully prepared a recombinant protein of *Drosophila* Rh7 in mammalian cultured cells. *Drosophila* Rh7 bound both 11-*cis*-retinal and 11-*cis*-3-hydroxyretinal to form photo-pigments which can absorb UV light. Irradiation with UV light caused formation of a visible-light absorbing metarhodopsin that activated Gq-type of G protein. This state could be photoconverted back to the original state and, thus Rh7 is a Gq-coupled bistable pigment. Interestingly, Rh7 (lambda max = 350 nm) exhibited an unusual broad spectrum with a longer wavelength tail reaching 500 nm, whose shape is like a composite of spectra of two pigments. In contrast, replacement of lysine at position 90 with glutamic acid caused the formation of a normal-shaped absorption spectrum with maximum at 450 nm. Therefore, Rh7 is a unique photo-sensor that can cover a wide wavelength region by a single pigment to contribute to non-visual photoreception.

## Introduction

The visual system of the fruit fly *Drosophila melanogaster* has been extensively studied and is one of the model systems for invertebrate vision^1–3^. The genome of *D*. *melanogaster* contains seven genes of rhodopsins (Rh1 to Rh7)^4^ for light reception, and six of them (Rh1-6) are expressed in different sets of photoreceptor cells in the retinae^5^. Rh7 is the most recently identified rhodopsin found by the fly genome project^6^. A recent paper about the analysis of Rh7 in fruit fly has revealed that Rh7 is distributed in a subset of neurons in the brain and functions in circadian photoentrainment^7^. Therefore, Rh7 is a unique rhodopsin specialized not for visual function but for non-visual photoreception in the brain.

Investigating the molecular properties of a rhodopsin is a critical step to elucidate the molecular basis of its physiological function. In fact, many vertebrate rhodopsins that function in visual and non-visual photoreception have been expressed in mammalian cultured cells to facilitate analysis of their spectroscopic and biochemical properties, including spectral sensitivity, along with their physiological functions. However, the expression levels of invertebrate rhodopsins, including fruit fly rhodopsins, in cultured cells are quite low. Thus, the absorption spectra of fruit fly rhodopsins were historically investigated by ectopic expression of rhodopsin genes in ommatidia R1–R6 cells of the ninaE mutant of fruit fly^8^. The electrical responses of photoreceptor cells containing rhodopsins other than Rh1 also showed that the expressed rhodopsins are coupled with Gq-type of G protein. However, preparation of recombinant proteins in cultured cells is one of the essential steps to evaluate the detailed molecular properties of rhodopsins by biochemical and biophysical methods.

In the present report, we describe expression of recombinant *Drosophila* Rh7 in mammalian cultured cells. After optimizing expression procedures, we successfully obtained recombinant Rh7 protein reconstituted with both 11-*cis*-retinal and 11-*cis*-3-hydroxyretinal. Then we improved the purification yield of Rh7 by replacement of its C-terminal region with the corresponding region of honeybee (*Apis cerane*) UV light-sensitive opsin. The evaluation of the molecular properties of Rh7 indicated that it is a UV light-sensitive pigment with bistable character. However, it exhibited an unusual broad absorption band that was not observed previously, suggesting that this property is one of the important clues to the elucidation of the function of Rh7.

## Results

### Heterologous expression of Rh7

**Figure 1:**
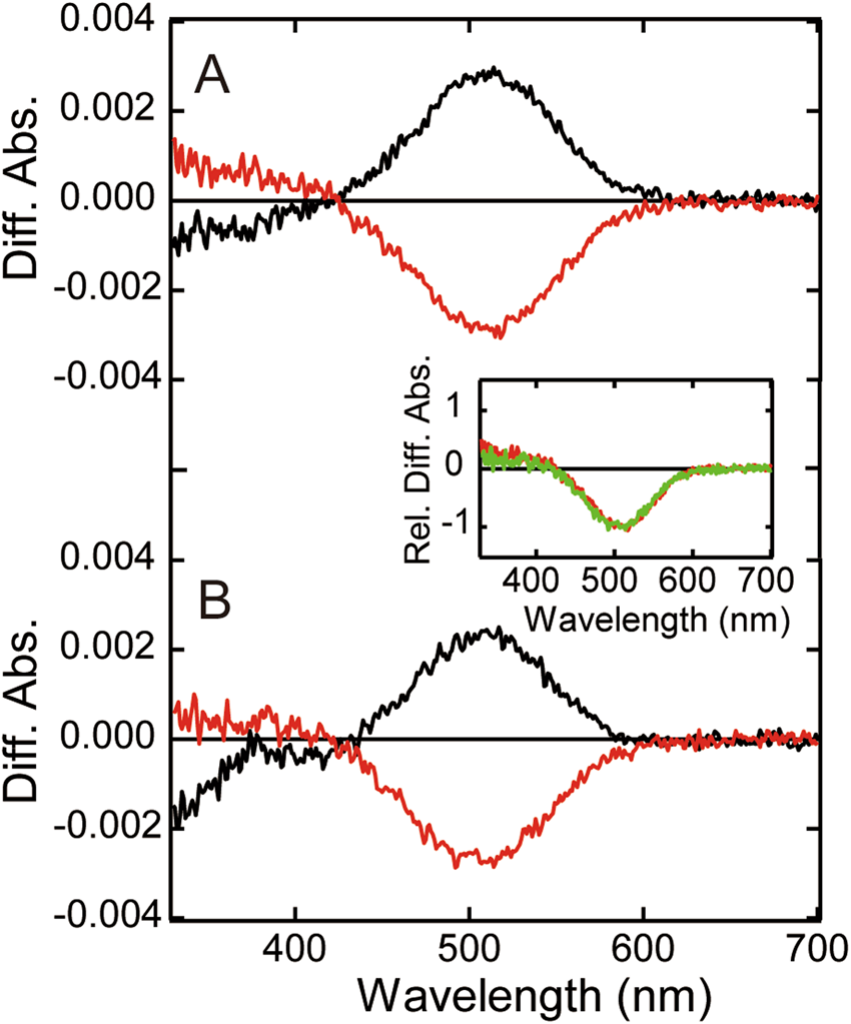
Photoreactions of DM-solubilized *Drosophila* Rh7. Difference spectra of DM-solubilized Rh7 regenerated with 11-*cis*-retinal (**A**) and 11-*cis*-3-hydroxyretinal (**B**) are shown. The black curve was calculated by subtracting the spectrum before irradiation from that after UV light irradiation. The red curve was calculated by subtracting the spectrum after UV light irradiation from that after subsequent yellow light (>500 nm) irradiation. Inset shows the superposition of difference spectra shown by red curves in (**A**) and (**B**). Spectra of samples regenerated with 11-*cis*-retinal (red curve) and 11-*cis*-3-hydroxyretinal (green curve) were normalized to be ~1.0 at the negative maximum.

*Drosophila* Rh7 was heterologously expressed in HEK293T cells and regenerated with 11-*cis*-retinal or 11-*cis*-3-hydroxyretinal, the latter of which is thought to be the native chromophore of *Drosophila* rhodopsins in the eyes^9, 10^. The regenerated pigments were solubilized with a detergent, n-dodecyl-β-D-maltoside (DM). The phylogenetic analysis of insect opsins indicated that Rh7 is closely related to short wavelength and UV light-sensitive rhodopsin groups (Fig. S1). Thus, we measured the spectral changes of the solubilized pigments induced by UV light irradiation. Figure 1A and B show difference spectra before and after photoreactions of pigments regenerated with 11-*cis*-retinal and 11-*cis*-3-hydroxyretinal, respectively. UV light irradiation of these pigments resulted in a decrease of absorbance in the UV region (~300–400 nm) and an increase in absorbance in the visible region (~500 nm) (black curve). Subsequent yellow light (>500 nm) irradiation caused a decrease of absorbance in the visible region with a concurrent increase in absorbance in the UV region (red curve). This photo-reversibility is commonly observed in several other UV light-sensitive opsins, such as insect visual opsin^8, 11^, vertebrate non-visual opsin parapinopsin^12^, and vertebrate Opn5^13–15^, and contrasts with the photoreaction of vertebrate UV light-sensitive cone pigments^16^. Superposition of the difference spectra calculated from the spectra measured before and after yellow light irradiation indicated that the spectra of Rh7 pigments regenerated with retinal and 3-hydroxyretinal are very similar to each other (Fig. 1 inset).

### Photoreaction of purified *Drosophila* Rh7

**Figure 2:**
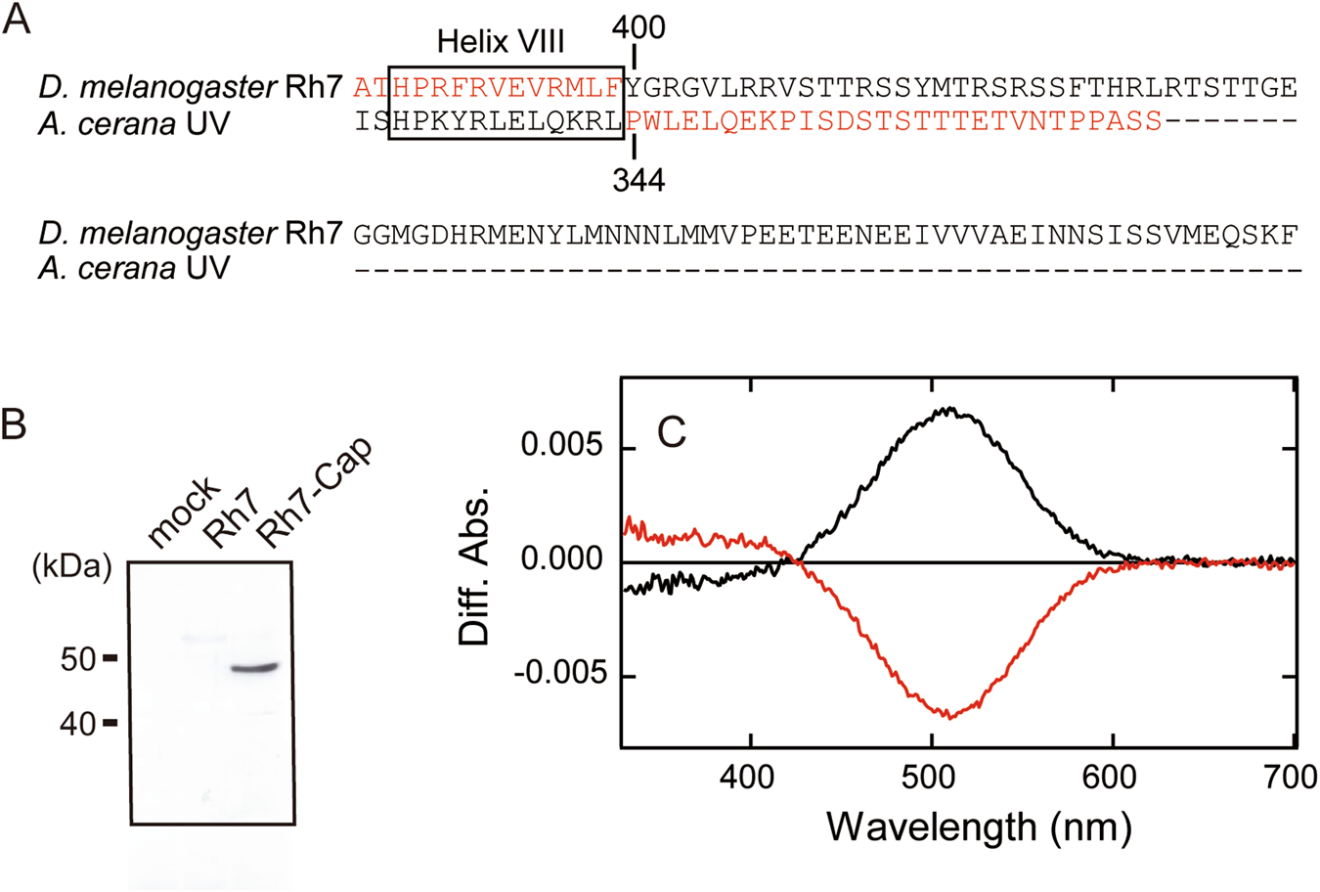
Construction of *Drosophila* Rh7-Cap. (**A**) Alignment of C-terminal region of *Drosophila* Rh7 and *A*. *cerana* UV light-sensitive visual opsin. The location of Helix VIII in the crystal structure of squid rhodopsin^46^ is indicated by a box. The amino acid sequence from position 400 to the C-terminal end of Rh7 was substituted with the corresponding amino acid sequence (from position 344 to the C-terminal end) of *A*. *cerana* UV light-sensitive visual opsin. (**B**) Western blot analysis against the C-terminal rho1D4 epitope tag. A clear signal was detected in Rh7-Cap-transfected cells, but not in mock- or Rh7 (full-length)- transfected cells. (**C**) Photoreaction of Rh7-Cap regenerated with 11-*cis*-retinal. The black curve was calculated by subtracting the spectrum before irradiation from that after UV light irradiation. The red curve was calculated by subtracting the spectrum after UV light irradiation from that after subsequent yellow light (>500 nm) irradiation.

In order to accurately determine the absorption spectrum and investigate the molecular properties of Rh7, we tried to purify the recombinant protein of Rh7 by affinity chromatography using an antibody (rho1D4) against the epitope tag inserted into the C-terminus. Unexpectedly, the absorption spectrum of the eluted sample contained no peaks in the visible or near UV regions (300~650 nm). In order to confirm the presence of the epitope tag in the sample, the DM-solubilized sample was subjected to western blotting using rho1D4 (Fig. 2B). There were no clear bands in the lane, suggesting that a posttranslational cleavage of the C-terminus had occurred and would hinder the affinity purification.

It was reported that the long C-terminus of squid rhodopsin was easily cleaved^17^. Therefore, based on the expectation that the relatively long C-terminus of Rh7 would be cleaved before the affinity column purification process, we substituted the C-terminal sequence of Rh7 with that of honeybee (*A*. *cerana*) UV light-sensitive visual opsin, which could be successfully purified by affinity chromatography using the epitope tag in the C-terminus^11^ (Fig. 2A). Hereafter we call this mutant protein Rh7-Cap. As expected, Rh7-Cap in the solubilized sample was clearly detected by western blotting using rho1D4 (Fig. 2B). Photoreactions of DM-solubilized Rh7-Cap reconstituted with 11-*cis*-retinal (Fig. 2C) were indistinguishable from that of wild-type Rh7 (Fig. 1A), suggesting that the substitution in the C-terminus caused no significant change in the absorption spectrum of Rh7.

**Figure 3:**
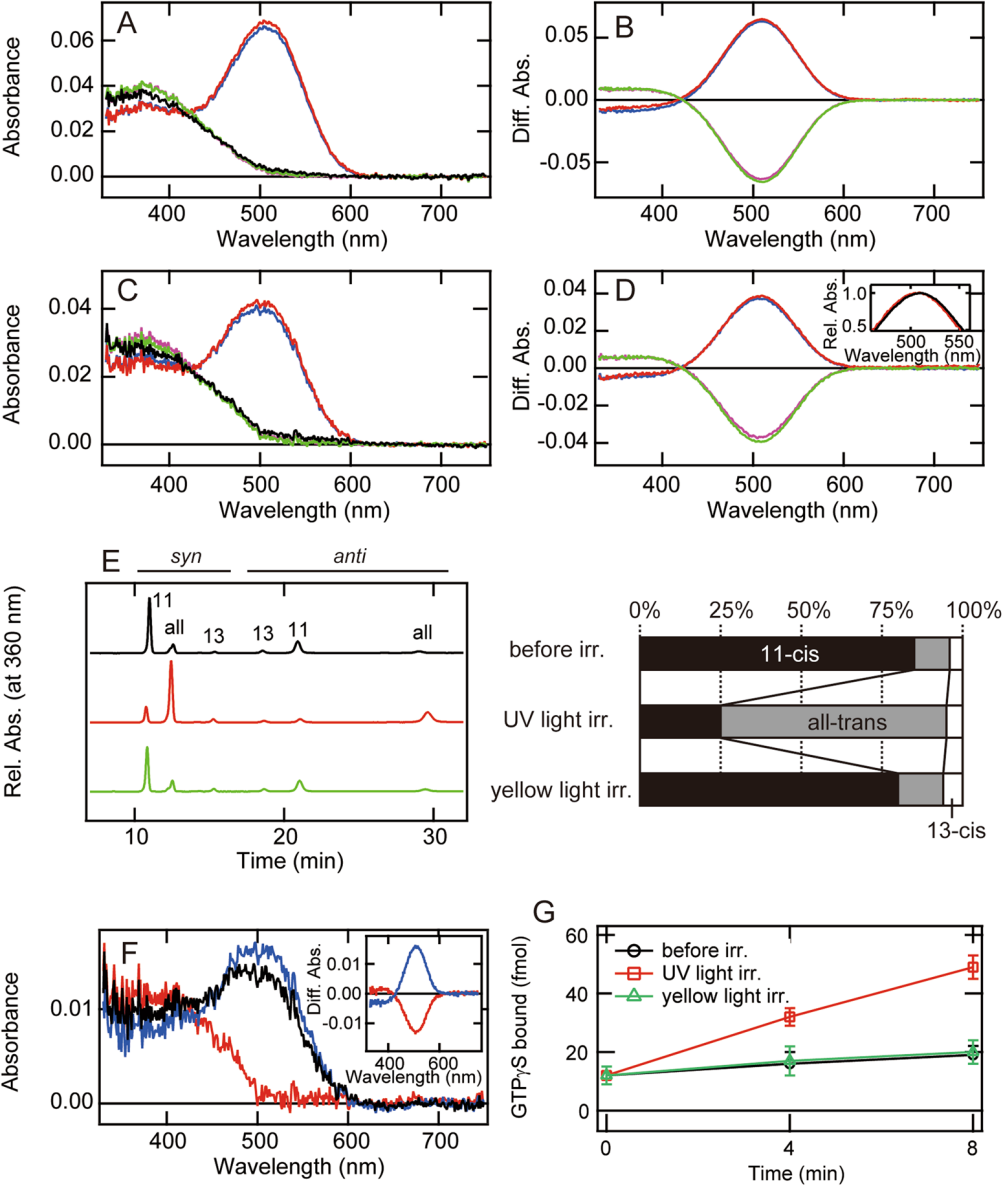
Molecular properties of purified *Drosophila* Rh7. (**A**–**D**) Absorption spectra of Rh7-Cap purified after it was regenerated with 11-*cis*-retinal (**A**,**B**) or 11-*cis*-3-hydroxyretinal (**C**,**D**). (**A**,**C**) Spectra were measured in the dark (black curve), after UV light irradiation (red curve), after subsequent yellow light (>500 nm) irradiation (green curve), after UV light re-irradiation (blue curve), and after yellow light re-irradiation (violet curve). (**B**,**D**) Difference spectra calculated based on the spectra in (**A**) or (**C**). The red curve was calculated by subtracting the spectrum before from that after UV light irradiation. The green curve was calculated by subtracting the spectrum after UV light irradiation from that after subsequent yellow light irradiation. The blue curve was calculated by subtracting the spectrum after yellow light irradiation from that after UV light re-irradiation. The violet curve was calculated by subtracting the spectrum after UV light re-irradiation from that after yellow light re-irradiation. The inset of (**D**) shows a comparison of the spectral properties of Rh7-Cap reconstituted with 11-*cis*-retinal (black curve) and 11-*cis*-3-hydroxyretinal (red curve). Difference spectra caused by UV light irradiation shown by red curve in (**B**) and (**D**) were normalized to be ~1.0 at the positive maxima. (**E**) Light-dependent retinal configuration changes of 11-*cis*-retinal bound Rh7-Cap. *Left-hand panel*, the retinal configurations were analyzed with HPLC after extraction of the chromophore as retinal oximes (*syn* and *anti* forms of 11-*cis*-, 13-*cis*-, and all-*trans*-retinal oximes). *Right-hand panel*, isomeric compositions of retinal before and after light irradiation. (**F**) Absorption spectra of Rh7-Cap purified after it was regenerated with all-*trans*-retinal. Spectra were measured in the dark (black curve), after yellow light (>500 nm) irradiation (red curve), and after subsequent UV light irradiation (blue curve). *Inset*, Difference spectra calculated by subtracting the spectrum before from that after yellow light irradiation (red curve) and the spectrum after yellow light irradiation from that after subsequent UV light irradiation (blue curve). (**G**) Time courses of G protein activation ability of Rh7-Cap reconstituted with 11-*cis*-retinal. Gq activation abilities were measured in the dark (black line), after UV light irradiation (red line), and after subsequent yellow light irradiation (green line). Data are presented as the means ± S.E. of three independent experiments.

Rh7-Cap regenerated with 11-*cis*-retinal was purified by affinity chromatography using the rho1D4 epitope tag (Figs 3A and S2A). The absorption spectrum of Rh7-Cap (black curve) exhibited a maximum (λ_max_) at about 375 nm, which is a longer wavelength than those of Rh3 and Rh4^8^, and an unusually broad absorbance tail extending up to 500 nm. UV light irradiation converted it to a visible light-absorbing state (λ_max_; 510 nm) with an extinction coefficient much larger than that of the original UV light-absorbing state (red curve). Subsequent yellow light irradiation (>500 nm) caused a spectral change that restored the original state (green curve). It should be noted that the spectrum recorded after yellow light irradiation (green curve) showed a slightly higher absorbance at the peak, suggesting that the purified pigment contained a small amount of the pigment having all-*trans*-retinal, which can convert back to the UV light-absorbing pigment having 11-*cis*-retinal (see below). Further irradiation with the UV light caused the formation of the state (blue curve) having a spectrum similar to that (red curve) obtained by the first irradiation of UV light, and re-irradiation with yellow light caused the formation of the state (violet curve) having an identical spectrum with that (green curve) obtained by the first yellow light irradiation. Thus, the light-dependent interconversion between the UV light-absorbing and visible light-absorbing states was repeatedly observed (Fig. 3B). These spectral changes are almost the same as those observed for the DM-solubilized sample (Fig. 2C), indicating that purification does not significantly alter the absorption spectra or photoreactions of Rh7-Cap. We also purified Rh7-Cap regenerated with 11-*cis*-3-hydroxyretinal. This pigment exhibited almost the same absorption spectra and photoreactions as those of Rh7-Cap containing 11-*cis*-retinal (Figs 3C,D and S2B), except that the visible light-absorbing state showed a slightly blue-shifted λ_max_ (506 nm) (Fig. 3D inset).

Next, we analyzed the change of the retinal configurations in *Drosophila* Rh7 reconstituted with 11-*cis*-retinal. Retinal chromophore was extracted as retinaloxime from purified Rh7-Cap samples before and after light irradiation and subjected to HPLC analysis (Fig. 3E). The extracted chromophore was mainly an 11-cis configuration with small amounts of all-trans and 13-cis configurations. UV light irradiation caused a decrease of 11-cis configuration with concurrent increase in all-trans configuration. Subsequent yellow light irradiation restored the original 11-cis configuration, which is consistent with the recovery of the absorption spectrum as shown in Fig. 3A and B. These results, together with the observed spectral changes, demonstrated that the UV light-absorbing state and the visible light-absorbing photoproduct contain 11-*cis*- and all-*trans*-retinals, respectively, and that light triggers cis-trans isomerization of the chromophore within the protein.

Many bistable opsins can directly bind both 11-*cis*- and all-*trans*-retinals^14, 18, 19^. Therefore, we also examined whether or not *Drosophila* Rh7 could bind to all-*trans*-retinal directly. We purified the recombinant protein after regeneration with all-*trans*-retinal and found that the recombinant protein exhibited λ_max_ in the visible region (Figs 3F and S2C, black curve). The amount of purified protein was lower than that obtained when the recombinant protein was reconstituted with 11-*cis*-retinal. Yellow light irradiation of the recombinant protein shifted the maximum to the UV region (red curve) and subsequent UV light irradiation restored the spectrum of the original state (blue curve), which can be explained by the light-dependent interconversion between the UV light-absorbing and visible light-absorbing states (Fig. 3F inset). These results indicated that *Drosophila* Rh7 has the ability to directly bind all-*trans*-retinal to form the photo-pigment.

### G protein activation by *Drosophila* Rh7

In the phylogenetic tree, *Drosophila* Rh7 belongs to Gq-coupled opsin group together with other *Drosophila* rhodopsins (Fig. S1). However, *Drosophila* Rh7 has a third cytoplasmic loop, an important G protein coupling region, 14–15 amino acids shorter than the third cytoplasmic loops of the other *Drosophila* rhodopsins^20, 21^, which suggests the possibility that *Drosophila* Rh7 has lost the G protein activation ability. Thus, we next investigated whether or not *Drosophila* Rh7 has the ability to couple with G protein (Fig. 3G). The dark state Rh7-Cap purified after reconstitution with 11-*cis*-retinal exhibited quite low Gq activation efficiency. UV light irradiation of the sample elevated the G protein activation efficiency, and subsequent yellow light irradiation decreased the efficiency. These results clearly indicated that *Drosophila* Rh7 can activate the Gq-type of G protein and that the visible light-absorbing species having all-*trans*-retinal is the active metarhodopsin state. In addition, the results suggested that the short third cytoplasmic loop of *Drosophila* Rh7, whose length is almost the same as that of bovine rhodopsin, maintains the sequence(s) crucial for G protein activation.

Because of low expression level and difficult purification of the recombinant proteins of the *Drosophila* rhodopsins including Rh7, we did not compare the Gq activation ability between Rh7 and other *Drosophila* rhodopsins. However, the recent report indicated that ectopic expression of Rh7 in ommatidia R1–R6 cells of ninaE mutant fly rescues the ERG response^7^. Thus, we speculate that Rh7 activity can be sufficient to lead to the photo-response in the cells.

### Spectral characterization of Rh7

**Figure 4:**
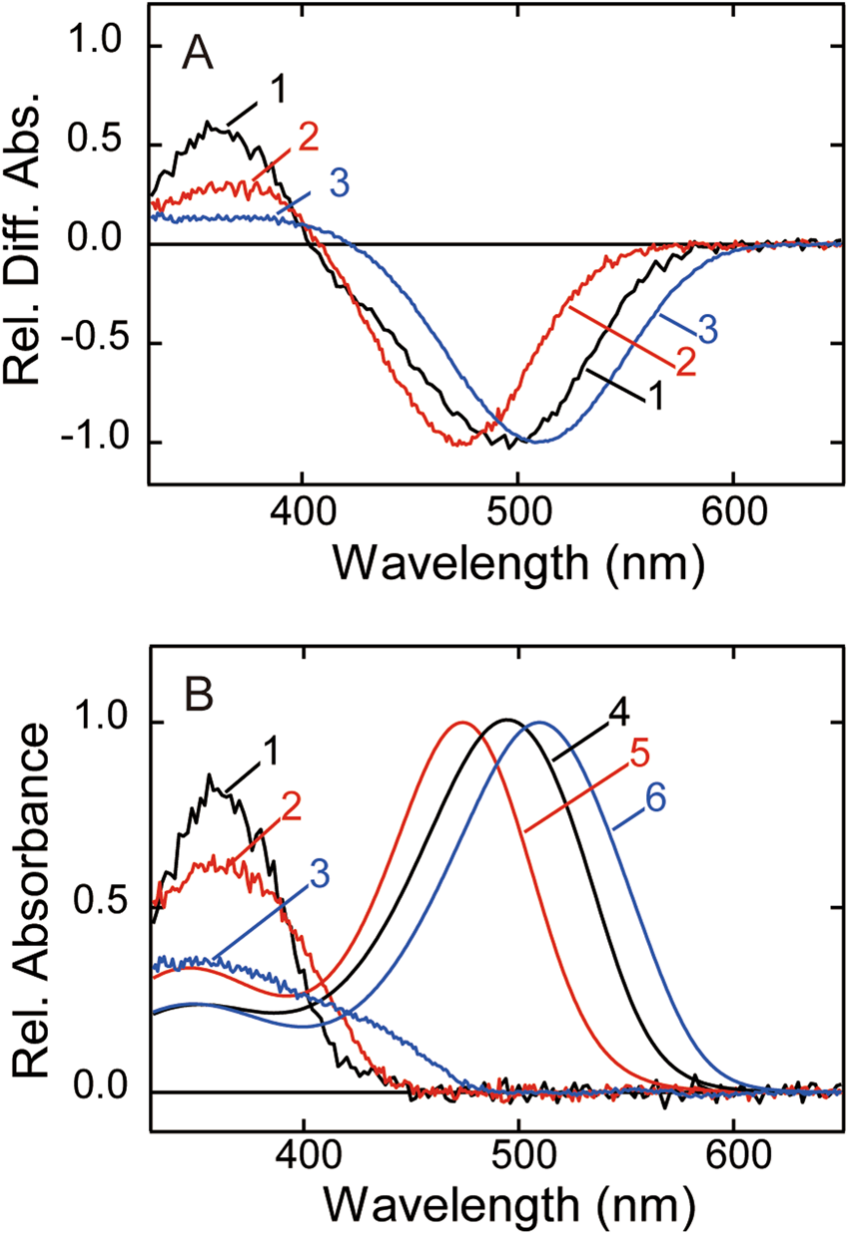
Spectral comparison of Rh7, parapinopsin, and Opn5m. (**A**) Spectral changes from all-*trans*- to 11-*cis*-retinal bound forms of lamprey parapinopsin (curve 1), chicken Opn5m (curve 2) and *Drosophila* Rh7-Cap (curve 3). Spectra were normalized to be ~1.0 at the negative maximum. (**B**) The calculated absorption spectra of three UV light-sensitive opsins (11-*cis*-retinal bound forms, curves 1–3) and their active states (all-*trans*-retinal bound forms, curves 4–6). Difference spectra of parapinopsin and Opn5m (curves 1 and 2 of (**A**)) were fitted with a template spectrum of bovine rhodopsin meta-I modeled by the Lamb and Govardovskii method^22, 23^ to calculate absorption spectra of all-*trans*-retinal bound forms (curves 4 and 5, respectively). Difference spectrum of Rh7 (curve 3 of (**A**)) was fitted with a template spectrum of squid retinochrome modeled by the Lamb and Govardovskii method to calculate the absorption spectrum of the all-*trans*-retinal bound form (curve 6). It should be noted that we calculated absorption spectra of three pigments by using the spectrum of squid retinochrome as a template; however, the base lines of spectra were not steady except for that of Rh7 (Fig. S3). Spectra of 11-*cis*-retinal bound forms of parapinopsin, Opn5m and Rh7 were determined by subtracting the difference spectra in (**A**) from the calculated spectra of all-*trans*-retinal bound forms (curves 1, 2 and 3, respectively).

As shown in Fig. 3B, Rh7 and its metarhodopsin can be photoconverted to each other upon absorption of UV and visible light, indicating that Rh7 is a bistable pigment. One of the characteristic properties of Rh7 is that the absorbance change in the UV region of the difference spectrum is extraordinarily small as compared to that in the visible region (a ratio of about 1 to 8). We compared the difference spectrum of Rh7 with those of other UV light-sensitive bistable pigments, lamprey parapinopsin^12^ and chicken Opn5m^14^ (Fig. 4A). In parapinopsin the difference absorbance in the UV region is about 0.7 times that in the visible region. Opn5m shows about half as large a difference absorbance in the UV region as that observed in parapinopsin, while it shows about 2 times higher difference absorbance there than Rh7. Thus, we calculated the absorption spectra of visible light-absorbing active states of Rh7, parapinopsin and Opn5m by the methods reported by Lamb^22^ and Govardovsky *et al*.^23^ using the absorption spectrum of bovine rhodopsin meta-I or squid retinochrome as a template^24, 25^. Figure 4B shows the calculated spectra of the native and metarhodopsin states of Rh7, parapinopsin and Opn5m.

Parapinopsin exhibits a normal absorption spectrum with λ_max_ at about 360 nm, while Opn5m shows a relatively broad absorption spectrum whose peak is at 360 nm, with smaller extinction coefficient. In contrast, Rh7 shows λ_max_ at about 350 nm and an unusually broad spectrum with a longer wavelength tail reaching 500 nm. Furthermore, the spectral shape of Rh7 is like a composite of the spectra of two pigments having maxima in the UV and blue regions. This unusual spectral shape could be a reflection of the chromophore environment or the chromophore structure in the chromophore-binding site of Rh7 (see Discussion).

**Figure 5:**
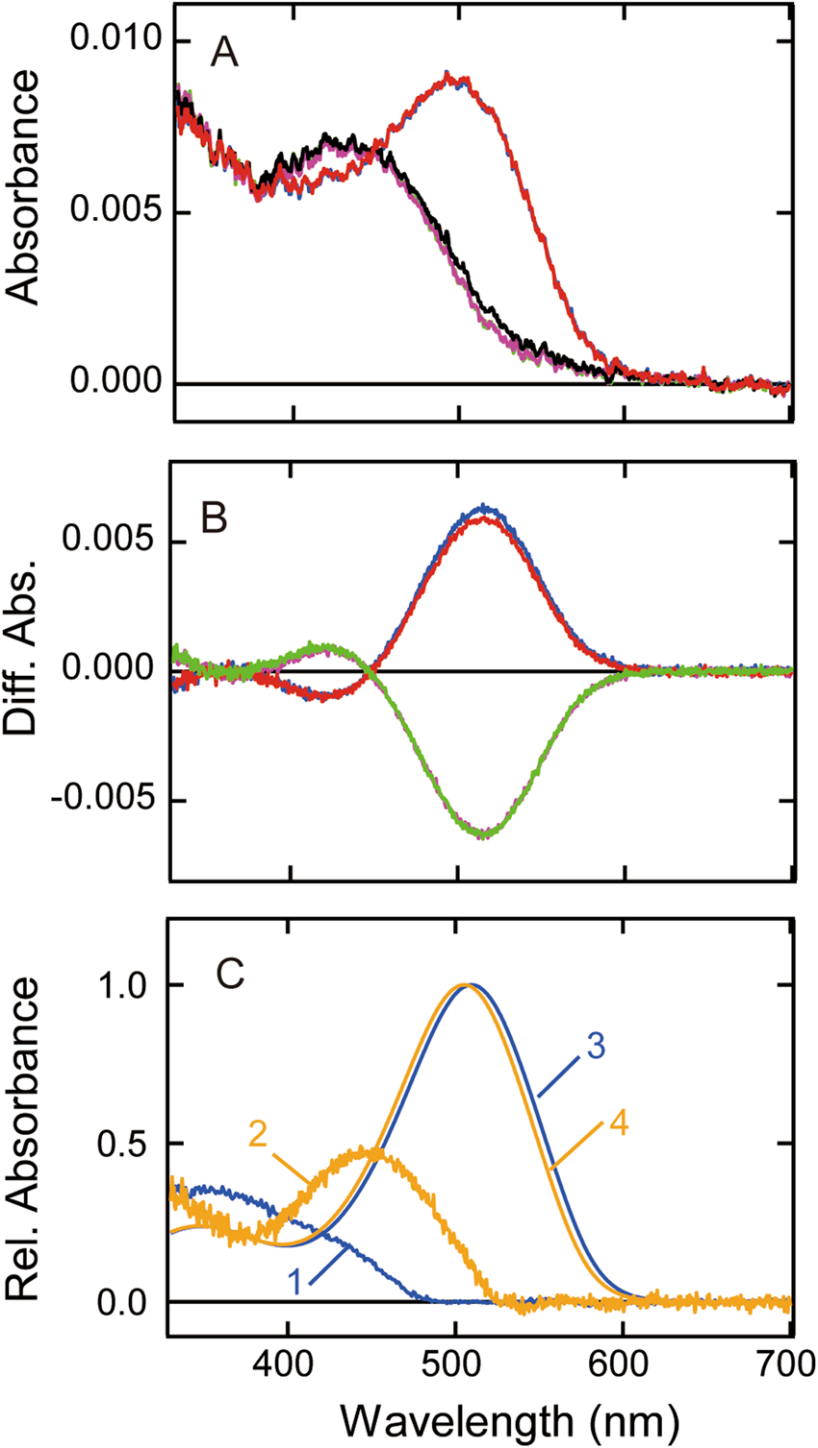
Spectral analyses of Rh7 K90E mutant. (**A**) Absorption spectra of Rh7-Cap K90E mutant. Absorption spectra of K90E mutant purified after it was regenerated with 11-*cis*-retinal were measured in the dark (black curve), after blue light (450 nm) irradiation (red curve), after subsequent orange light (>550 nm) irradiation (green curve), after blue light re-irradiation (blue curve), and after orange light re-irradiation (violet curve). (**B**) Difference spectra of Rh7-Cap K90E mutant calculated based on the spectra in (**A**). The red curve was calculated by subtracting the spectrum before irradiation from that after blue light irradiation. The green curve was calculated by subtracting the spectrum after blue light irradiation from that after subsequent orange light irradiation. The blue curve was calculated by subtracting the spectrum after orange light irradiation from that after blue light re-irradiation. The violet curve was calculated by subtracting the spectrum after blue light re-irradiation from that after orange light re-irradiation. (**C**) The calculated absorption spectra of Rh7-Cap K90E mutant. Difference spectrum of Rh7-Cap K90E mutant (green curve of (**B**)) was fitted with a template spectrum of squid retinochrome modeled by the Lamb and Govardovskii method to calculate the absorption spectrum of its all-*trans*-retinal bound form. Spectrum of 11-*cis*-retinal bound forms was determined by subtracting the difference spectrum in (**B**) from the calculated spectrum of all-*trans*-retinal bound form. Spectra of the dark (curve 2) and irradiated (curve 4) states of Rh7-Cap K90E mutant were compared with those of Rh7-Cap (curves 1 and 3).

Mutational analysis of avian UV light-sensitive cone pigments revealed that the cysteine residue at position 90 (in the bovine rhodopsin numbering system) is one of the determinants of UV light reception^15, 26^. On the other hand, the lysine residue at the same position in *Drosophila* UV light-sensitive rhodopsins is also responsible for their UV light receptions^27^. This lysine residue is well conserved not only in the UV light-sensitive rhodopsin groups of insects but also in the UV light-sensitive rhodopsins of jumping spiders^28^. *Drosophila* Rh7 also has a lysine residue, whereas the Rh7 of some insects has a glutamic acid residue at this position (Fig. S4). Therefore, we prepared the recombinant protein of K90E mutant reconstituted with 11-*cis*-retinal (Fig. 5A). The K90E mutant showed a normal shaped absorption spectrum with λ_max_ at about 450 nm (black curve). Blue (450 nm) and orange (>550 nm) light irradiations revealed a bistable character with a state having λ_max_ at about 500 nm (Fig. 5A,B). By simulation of the spectra using the same method as that applied to Rh7-Cap, we determined the λ_max_ (450 nm) of Rh7 K90E mutant (Fig. 5C). Thus, Lys90 is a determinant residue for the UV light reception with the unusual spectral shape of Rh7.

## Discussion

In the present study, we successfully expressed Rh7, the only thus-far uncharacterized rhodopsin encoded by the *D*. *melanogaster* genome, in mammalian cultured cells, and regenerated it as a photo-pigment by the incorporation of 11-*cis*-retinal or 11-*cis*-3-hydroxyretinal. Rh7 contains a long C-terminal tail that can be posttranslationally cleaved in cultured cells, making it difficult to obtain recombinant Rh7, but substitution of the C-terminus of Rh7 with that of honeybee UV light-sensitive visual opsin enabled the purification of Rh7. The expression yield of fly Rh7 in cultured cells was about 20-fold lower than that of bovine rhodopsin. In addition, it was more difficult to maintain the Rh7 pigment during the purification process. Thus, the collection yield of Rh7 after the purification procedures was about 50-fold lower than that of bovine rhodopsin. Using this purified Rh7-Cap containing 11-*cis*-retinal, we characterized the absorption spectrum, photoreactions and G protein activation of Rh7.

It has been reported that almost all of the retinal in the head of adult *D*. *melanogaster* is 3-hydroxyretinal^9^, but there have been no reports on the retinal species present in other regions of the body. Thus, the endogenous retinal chromophore of Rh7 may differ depending on the tissues where Rh7 is expressed. Our experiments clearly showed that Rh7 exhibited similar spectrum and photoreaction regardless of whether it had incorporation of retinal or 3-hydroxylretinal.

**Figure 6:**
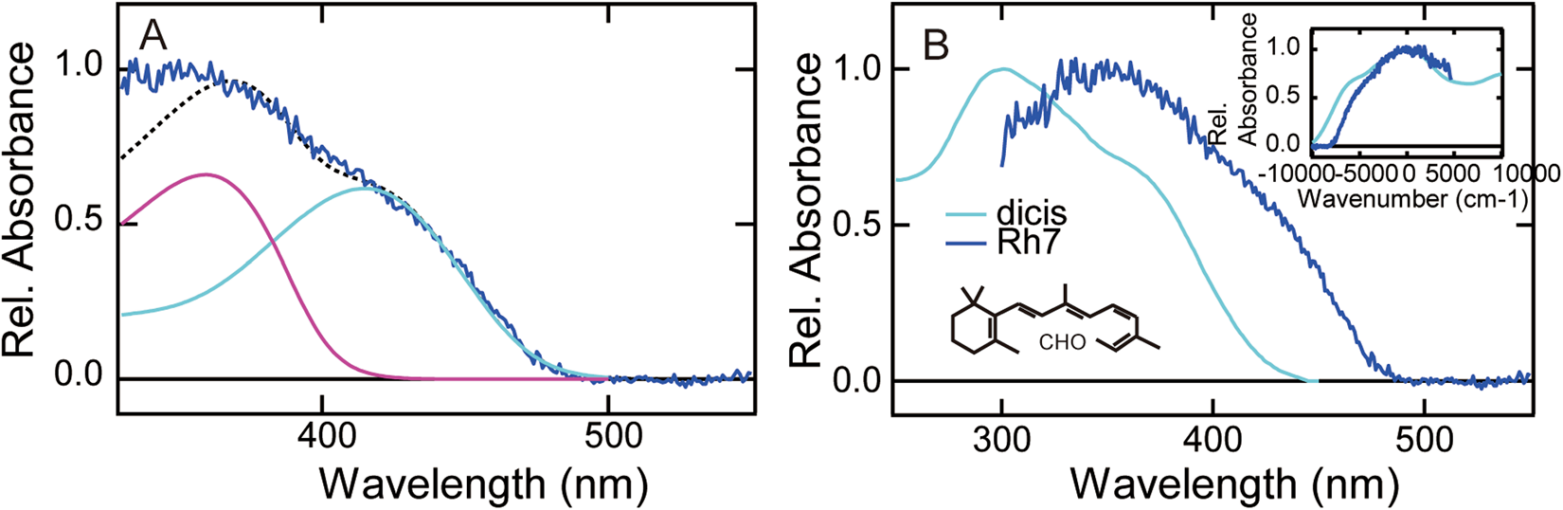
Spectral component analysis of Rh7. (**A**) Fitting of absorption spectrum of Rh7-Cap (blue curve) with two template spectra modeled by the Lamb and Govardovskii method (dashed black curve). λ_max_ of two template spectra are 415 nm (cyan curve) and 360 nm (magenta curve). (**B**) Comparison of absorption spectra between purified Rh7-Cap (blue curve) and 11,13-*dicis*-retinal in EPA (cyan curve). The structure of 11,13-*dicis*-retinal is shown in the graph. Inset shows the absorption spectra of Rh7-Cap and 11,13-*dicis*-retinal plotted against the wavenumber.

The most unique character of Rh7 is the shape of its absorption spectrum in the 11-*cis*-retinal bound state. As shown in Fig. 4B and C, Rh7 shows λ_max_ in the UV region but shows an unusually broad spectrum with a longer wavelength tail extending up to 500 nm. Therefore, the Rh7 spectrum seems to be a composite of two spectra showing λ_max_ in the UV and visible regions and is considerably different in shape from those of so far investigated UV light-absorbing pigments such as UV cone pigment, parapinopsin and Opn5m. In fact, the spectrum of Rh7 can be fitted with two template spectra of 11-*cis*-retinal bound pigments^22, 23^ having λ_max_ at 360 and 415 nm (Fig. 6A). To examine whether or not Rh7 is a composite of two states having different absorption spectra, we irradiated Rh7 with 450 nm light that was mainly absorbed by the longer wavelength region of the Rh7 spectrum. However, we did not detect any difference in spectral change between irradiations by 450 nm light and by UV light (Figs 3B and S5). We also examined the pH-dependent spectral shift of Rh7 but we did not observe any changes of the spectrum (Fig. S6). These results strongly suggested that the unique absorption spectrum of Rh7 is not due to a composite of two states but rather is due to the specific interaction between retinal chromophore and nearby protein. A spectral shape similar to that of Rh7 was previously observed in some of the isomers of retinal. Figure 6B shows the absorption spectrum of 11,13-*dicis*-retinal solubilized in organic solution (EPA)^29^. The specific shape of the absorption spectrum of 11,13-*dicis*-retinal is derived from its circular structure (Inset of Fig. 6B), so that the oscillator strength of the transition to the first excited state is lower than that to the second excited state. It is inferred that the crystal structure of 11-*cis*-retinal, which is in an 11-cis, 12s-cis conformation, could show an absorption spectrum similar to those of 11,13-*dicis*- and 9,11,13-*tricis*-retinals in solution^29^. Therefore, we propose another possibility, namely, that the chromophore of Rh7 is in a circular shape (12s-cis conformation) similar to that of the crystal structure of 11-*cis*-retinal, so that the absorption spectrum of Rh7 shows a unique shape due to the large oscillator strength of the transition to the second excited state. Further spectroscopic analysis is needed to elucidate the mechanism underlying the spectral shape of Rh7.

*Drosophila* Rh7 has a characteristic protein feature, a longer C-terminal region than other *Drosophila* rhodopsins, which is shared across the Rh7 group (Fig. 2A). The genomic analysis of “Dark-Fly”, which has been maintained in constant dark conditions for 57 years, showed that among the seven rhodopsin genes, a mutation occurred only in the Rh7 gene, which led to the truncation of the C-terminal 21 amino acids of Rh7 in the fly^20^. We could not evaluate the functional importance of the truncated C-terminus in *Drosophila* Rh7, because we could not succeed in purifying the recombinant protein having the native C-terminus. Further analysis of “Dark-Fly” will uncover the physiological significance of the truncated C-terminus of *Drosophila* Rh7, which may be related to the adaptation to a dark environment.

The phylogenetic tree of opsins showed that insect visual opsins belong to the Gq-coupled opsin group together with molluscan rhodopsin and vertebrate Opn4^30–32^ and are classified into three groups correlating to their spectral sensitivity: a long wavelength light-sensitive group (including *D*. *melanogaster* Rh1, Rh2 and Rh6), a short wavelength light-sensitive one (including *D*. *melanogaster* Rh5), and a UV light-sensitive one (including *D*. *melanogaster* Rh3 and Rh4)^33^. In addition, Rh7 genes have been identified from many insect genomes and form an independent group^21, 34^. The phylogenetic analysis of insect opsins showed that the Rh7 group was clustered with short wavelength light-sensitive and UV light-sensitive opsin groups and was diversified before branching of these two visual opsin groups (Fig. S1). Comparison of the amino acid sequences reveals that most members of the UV light-sensitive opsin group and Rh7 group have a lysine residue at position 90 (Fig. S4), which can account for the UV light reception of *Drosophila* Rh7 (Fig. 5A–C). Recently, analysis of the electroretinogram of transgenic flies expressing mosquito (*Aedes aegypti*) Rh7 (Aaop10) in the visual cells showed that visible light, not UV light, irradiation triggered the photoresponse^35^. This suggests that *D*. *melanogaster* and *A*. *aegypti* Rh7 share the Gq coupling property, although these opsins exhibit different spectral sensitivity. This visible light-sensitive *A*. *aegypti* Rh7 contains a glutamic acid residue instead of a lysine residue at position 90 (Fig. S4). The shift of the spectrum from the UV to visible region by the K90E mutation of *Drosophila* Rh7 (Fig. 5A) can explain the spectral tuning between *D*. *melanogaster* and *A*. *aegypti* Rh7s. Recent accumulation of genomic information in Arthropoda showed that Rh7 genes can be found not only in Hexapoda (insects), but also in water flea (*Daphnia pulex*) in Crustacea, and in horseshoe crab (*Limulus polyphemus*) and spider mite (*Tetranychus urticae*) in Chelicerata (Fig. S1). *D*. *pulex*, *L*. *polyphemus*, and *T*. *urticae* have multiple Rh7 genes, some of which have a lysine residue at position 90 and others of which have different residues at this position (Fig. S4). Thus, we speculate that these animals have both UV light-sensitive and visible light-sensitive Rh7.

It has been reported that visible light-sensitive *A*. *aegypti* Rh7 is distributed in a subset of UV light-sensitive retinal photoreceptor cells together with UV light-sensitive visual opsin^35^. This coexpression suggests the potential of Rh7 to modulate the visual process in *A*. *aegypti*. However, comparison of the mRNA expression of *Drosophila* rhodopsins showed that Rh7 has a much lower expression level than other rhodopsins in the photoreceptor cells of eyes^36^. *Drosophila* Rh7 has a uniquely broad absorption spectrum, with the wavelength range expanded from the UV region to the violet and blue regions. Thus, Rh7 covers a broad wavelength range by a single pigment at the expense of photosensitivity, which can be advantageous to work as a circadian photoreceptor protein in the brain of *D*. *melanogaster* ^7^. These differences between *D*. *melanogaster* and *A*. *aegypti* indicate the possibility that the Rh7 group can regulate both visual and non-visual functions depending on the molecular properties and the tissue distribution. Further evaluation of Rh7 from a variety of arthropods will reveal the diversity of Rh7-triggered physiological functions.

## Methods

### Preparation of purified Rh7

The cDNA clone of *D*. *melanogaster* Rh7 (NCBI accession number; AY060299) was obtained from FlyBase (http://flybase.org/). The cDNA of Rh7-Cap was generated by substituting 84 amino acids in the C-terminus of Rh7 with the corresponding sequence (28 amino acids) of honeybee (*A*. *cerana*) UV light-sensitive visual opsin (AB355816)^11^ using an In-Fusion cloning kit (Clontech) (Fig. 2A). The cDNA of Rh7-Cap K90E mutant was also constructed using an In-Fusion cloning kit according to the manufacturer’s instructions. The cDNAs were tagged by the epitope sequence of anti-bovine rhodopsin monoclonal antibody rho1D4 (ETSQVAPA) at the C-terminus and introduced into mammalian expression vector pCAGGS^37^. The plasmids were transfected into the HEK293T cell line using the calcium phosphate method. After incubation for 2 days, the cells were collected and incubated with 11-*cis*-retinal or 11-*cis*-3-hydroxyretinal (racemic mixture of 3R/3S enantiomers)^38^ overnight at 4 °C to regenerate photo-pigments. The reconstituted pigments were extracted with 1% DM in buffer A (50 mM HEPES, 140 mM NaCl, and 3 mM MgCl_2_, pH 6.5) and purified by adsorption on rho1D4-conjugated agarose. Purified pigments were eluted with buffer B (0.3 mg/mL synthetic peptide corresponding to the rho1D4 epitope sequence, 0.02% DM, 50 mM HEPES, 140 mM NaCl, and 3 mM MgCl_2_, pH 6.5). For spectroscopic experiments of solubilized samples without purification, collected cells reconstituted with 11-*cis*-retinal or 11-*cis*-3-hydroxyretinal were incubated with 20 mM or 5 mM hydroxylamine, respectively, for 2 hours to remove the random Schiff base formation. After washing three times with buffer A, the reconstituted pigments were extracted with 1% DM in buffer A.

### Spectrophotometry and high performance liquid chromatography (HPLC) analysis

Absorption spectra were measured using a Shimadzu UV-2400 or Hitachi U-4100 spectrophotometer at 0 °C. For irradiation, light from a 1-kW tungsten lamp (Master HILUX-HR, Rikagaku) which had been passed through a UV-D35 glass filter, a BP450 band-pass filter, a Y52 cutoff filter, an O57 cutoff filter or an O58 cutoff filter was used. The samples were irradiated until they reached photosteady states. Absorption spectra of the two states of Rh7-Cap and Rh7-Cap K90E mutant were calculated using the methods previously described^14^. Briefly, the spectral region at longer wavelengths of the difference spectrum calculated by subtracting the spectrum before irradiation from that after yellow light irradiation was best-fitted with a template spectrum of bovine rhodopsin meta-I or squid retinochrome which was modeled by previous studies^22, 23^. This was considered to be the spectrum of the irradiated state. The absorption spectrum of the original state was then calculated by adding the spectrum of the irradiated state to the difference spectrum. The chromophore configurations of each sample were analyzed by HPLC (LC-10AT VP; Shimadzu) as described previously^39^. The chromophores were extracted from Rh7 samples as retinaloximes by the addition of hydroxylamine and applied to a silica column (150 × 6 mm, A-012-3; YMC). The solvent was composed of 98.8% benzene, 1.0% diethyl ether, and 0.2% 2-propanol. HPLC patterns were obtained by monitoring the absorbance at 360 nm.

### G protein activation assay

The purified mouse Gqα complexed with β1γ2 which was expressed in Sf9 cells was kindly provided by Prof. T. Doi (Kyoto University)^40^. The comparison of the amino acid sequences between mouse and *D*. *melanogaster* Gqα shows that sequence similarity is about 92%, and notably, a five amino acid sequence in the C-terminus of Gα, which is the determinant region for G protein subtype specificity^41^, is identical. Thus, we utilized mouse Gqα as equivalent to *D*. *melanogaster* Gqα. A radionucleotide filter-binding assay, which measures GDP/GTPγS exchange by G protein, was performed as described previously^14^. All procedures were carried out at 0 °C. The assay mixture consisted of 50 mM HEPES (pH 7.0), 140 mM NaCl, 5 mM MgCl_2_, 1 mM DTT, 0.01% DM, 100 nM [^35^S]GTPγS and 2 μM GDP. Purified Rh7-Cap (final concentration: 100 nM) was mixed with G protein solution (final concentration: 100 nM) and was kept in the dark or irradiated with UV light for 1 min or with subsequent yellow light (>500 nm) for 1 min. After irradiation, the GDP/GTPγS exchange reaction was initiated by the addition of [^35^S]GTPγS solution to the mixture of the pigment and G protein. After incubation for the indicated time in the dark, an aliquot (20 μL) was removed from the sample, mixed with 200 μL of stop solution (20 mM Tris/Cl (pH 7.4), 100 mM NaCl, 25 mM MgCl_2_, 1 μM GTPγS and 2 μM GDP), and immediately filtered through a nitrocellulose membrane to trap [^35^S]GTPγS bound to G proteins. The amount of bound [^35^S]GTPγS was quantitated by assaying the membrane with a liquid scintillation counter (Tri-Carb 2910 TR; PerkinElmer).

### Western blot analysis

HEK293T cell membranes expressing Rh7 and Rh7-Cap were solubilized with DM, subjected to SDS-PAGE and then transferred to a PVDF membrane. The transferred proteins were subjected to western blot analysis using the monoclonal antibody rho1D4. Immunoreactive proteins were detected by the avidin biotin complex (ABC) method and visualized with a horseradish peroxidase-diaminobenzidine (DAB) reaction.

### Molecular phylogenetic analysis of opsins

The molecular phylogenetic tree was inferred using a maximum likelihood tree search algorithm, RAxML^42^, with the WAG model^43^ of amino acid substitution. The evolutionary rate heterogeneity among sites was also taken into account, assuming the discrete gamma model of Yang^44^. In the phylogenetic tree analysis, two molluscan Gq-coupled rhodopsins and two vertebrate Opn4 were included as putative outgroups of 100 arthropod opsins and a velvet worm (*Euperipatoides kanangrensis*) opsin. The amino acid sequences of these 105 opsins were aligned using the MAFFT program^45^ with manual curation, and 250 highly homologous sites without gap sites were selected for the tree inference.

## Electronic supplementary material

Supplementary information
