# Supplementary material for: Drosophila melanogaster rhodopsin Rh7 is a UV-to-visible light sensor with an extraordinarily broad absorption spectrum
Source: Sci Rep. 2017 Aug 4;7:7349. doi: 10.1038/s41598-017-07461-9 (PMC5544684; doi:10.1038/s41598-017-07461-9)
Supplement: Supplementary file 1 — Supplementary information [file 41598_2017_7461_MOESM1_ESM.pdf]

Supplementary Informatiuon

Drosophila melanogaster rhodopsin Rh7 is a UV-to-visible light sensor with an extraordinarily broad absorption spectrum

Kazumi Sakai[1†], Kei Tsutsui[1†], Takahiro Yamashita[1], Naoyuki Iwabe[1], Keisuke Takahashi[1], Akimori Wada[2] and Yoshinori Shichida[1*]

[1]Department of Biophysics, Graduate School of Science, Kyoto University, Kyoto 606-8502, Japan and [2]Department of Organic Chemistry for Life Science, Kobe Pharmaceutical University, Kobe 658-8558, Japan.

[†]These authors contributed equally to this work.

To whom correspondence should be addressed: Yoshinori Shichida, Department of Biophysics, Graduate School of Science, Kyoto University, Kyoto 606-8502, Japan.　E-mail: shichida@rh.biophys.kyoto-u.ac.jp

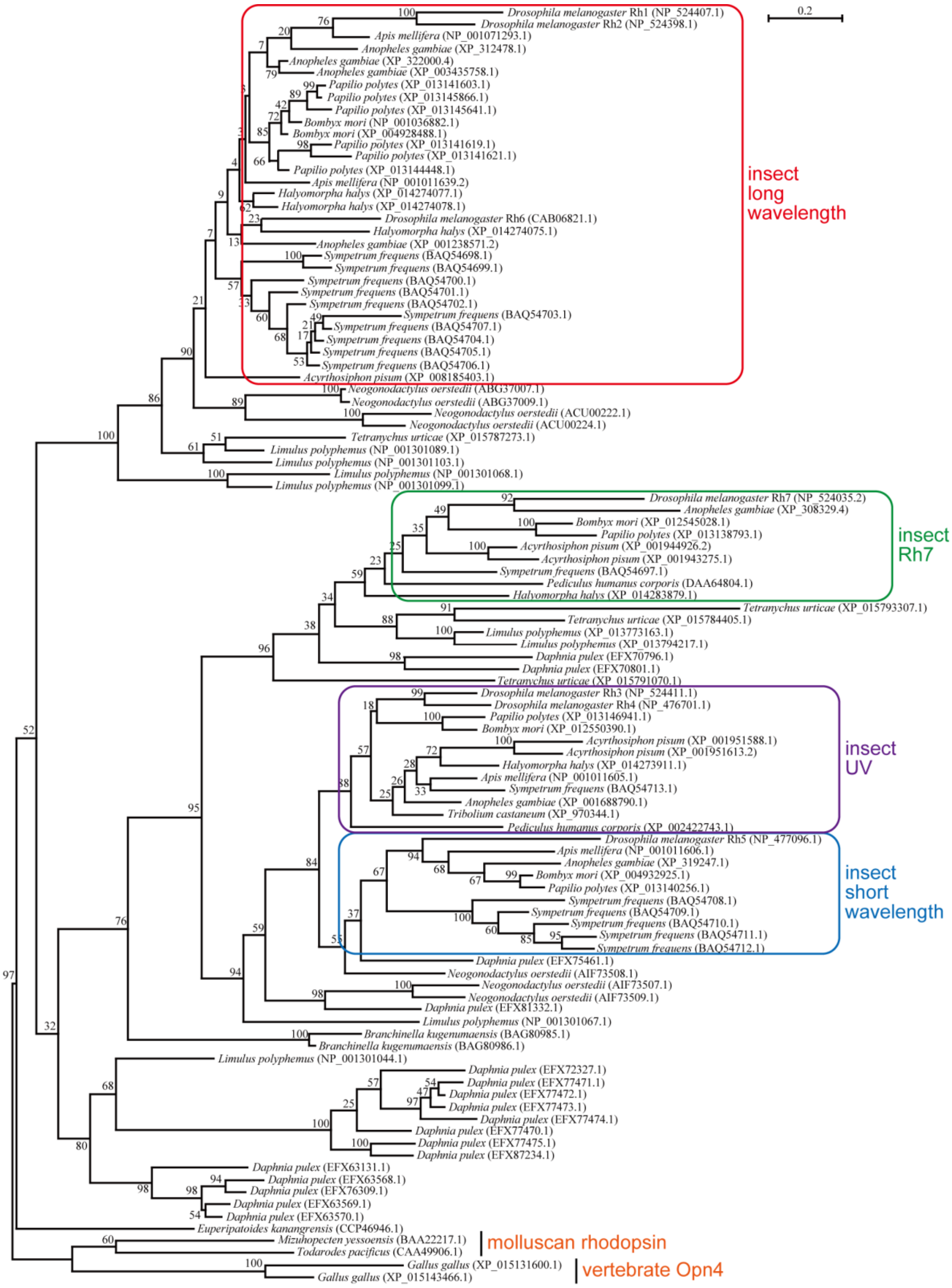

Fig. S1 Phylogenetic relationship of insect visual opsins and Rh7.

The amino acid sequences of 105 opsins were aligned using MAFFT [1] and the

molecular phylogenetic tree was inferred using RAxML version 8 [2], a maximum likelihood tree search program, using the WAG model [3] and Yang's discrete gamma model [4] with an optimized shape parameter alpha of 1.02. An unambiguous alignment of 250 amino acids in length excluding gaps was used for the tree inference. The numbers at each branch are the bootstrap probabilities obtained by 1,000 bootstrap resamplings [5]. NCBI accession numbers of opsins are shown in parentheses.

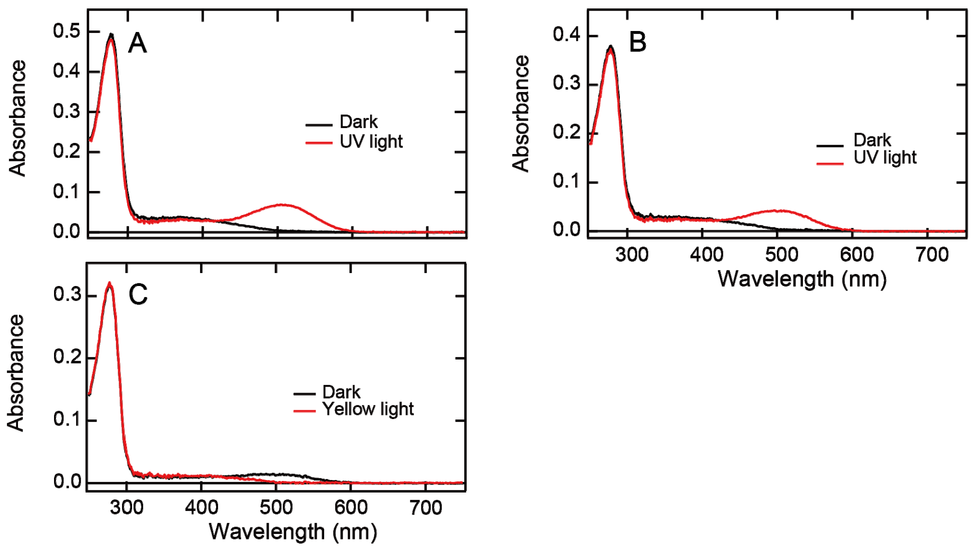

Fig. S2　Absorption spectra of purified *Drosophila* Rh7

(A, B) Absorption spectra of Rh7-Cap purified after regeneration with 11-*cis*-retinal (A) or 11-*cis*-3-hydroxyretrnal (B) (black curves).　Red curves show the spectra measured after UV light irradiation.　Optical purity of the sample (Abs280nm/Abs510nm) was calculated from the spectrum of the state containing all-trans chromophore, because Rh7-Cap regenerated with 11-cis chromophore exhibited an extraordinarily broad absorption spectrum.　The values were 7.0 (A) and 9.0 (B).　(C) Absorption spectrum of Rh7-Cap purified after regeneration with all-*trans*-retinal (black curve).　Red curve shows the spectrum measured after yellow light irradiation.　Optical purity was also calculated to be 19.5.

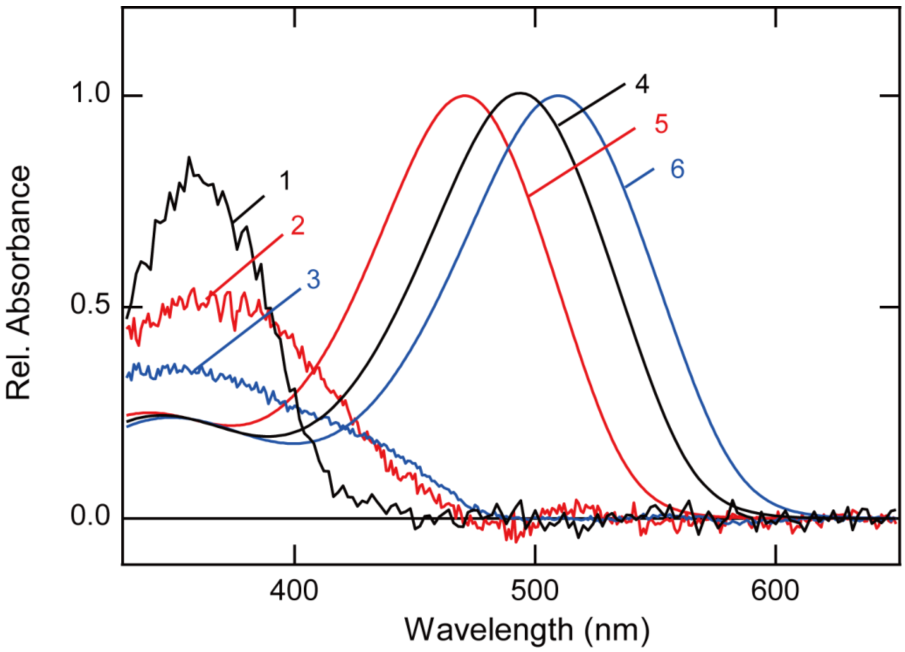

Fig. S3 Calculated absorption spectra of parapinopsin, Opn5m and Rh7 using the absorption spectrum of squid retinochrome as a template.

Difference spectra of parapinopsin, Opn5m and Rh7 (Fig. 4A) were fitted with a template spectrum of squid retinochrome modeled by Lamb and Govardovskii method to calculate absorption spectra of all-*trans*-retinal bound forms (curves 4, 5 and 6, respectively). Spectra of 11-*cis*-retinal bound forms of parapinopsin, Opn5m and Rh7 were determined by subtracting the difference spectra in Fig. 4A from the calculated spectra of all-*trans*-retinal bound forms (curves 1, 2 and 3, respectively).

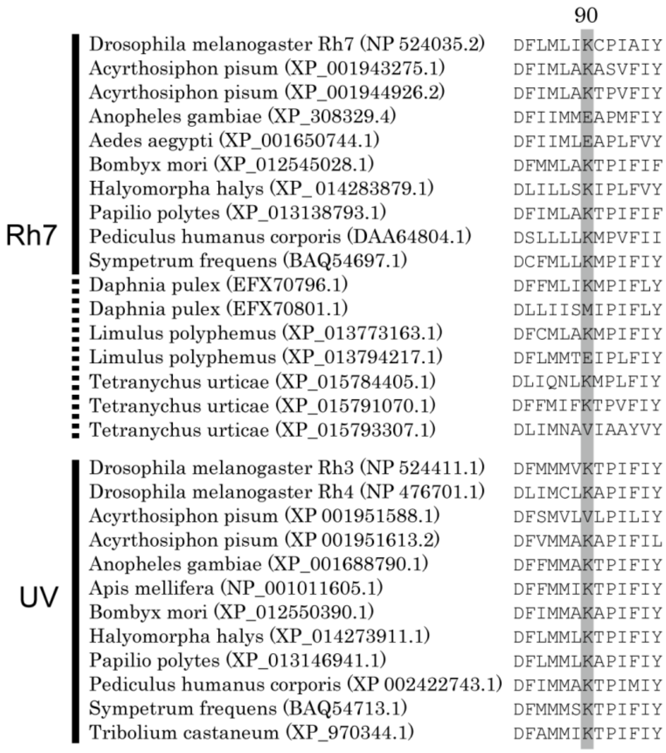

Fig. S4 Alignment of amino acid sequences of Rh7 group and insect UV light-sensitive opsin group around position 90.

The amino acid residues at position 90 (in the bovine rhodopsin numbering system) are boxed in gray.

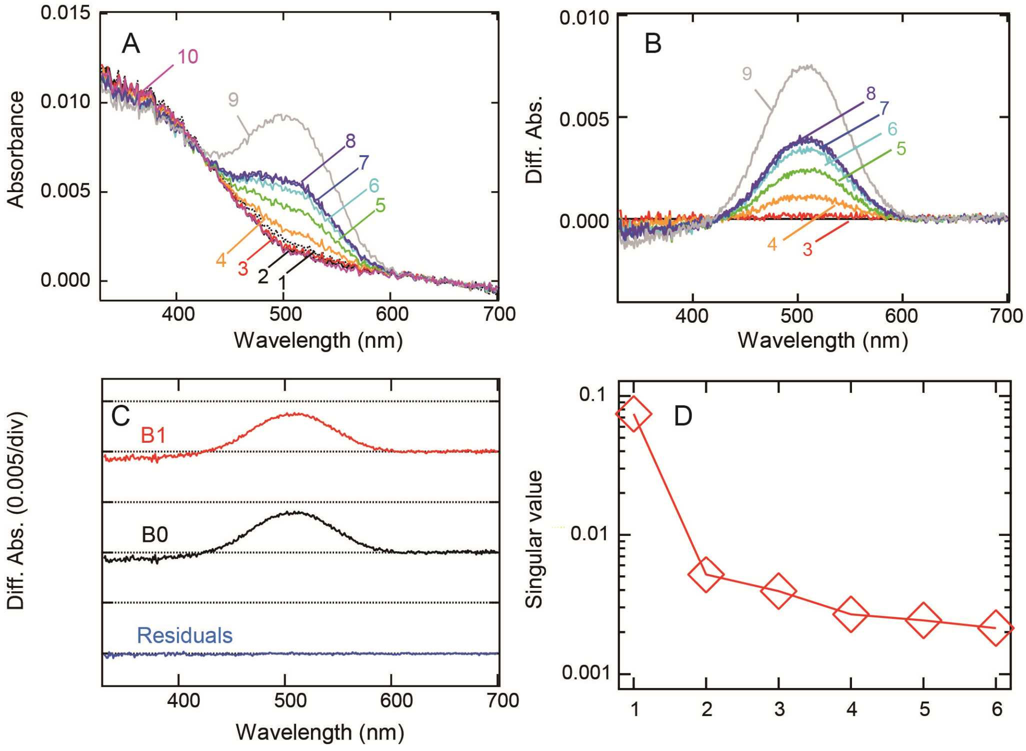

Fig. S5 Photoreactions of Rh7-Cap induced by blue light irradiation.

(A) Absorption spectra of Rh7-Cap reconstituted with 11-*cis*-retinal before and after blue light irradiation. Purified Rh7-Cap (dashed black curve, curve 1) was irradiated with >560 nm light (curve 2) to completely produce 11-*cis*-retinal bound form. Subsequently, it was irradiated with 450 nm light at 0 ºC to measure spectral changes (curves 3-8). Absorption spectra were recorded at the irradiation time of 5, 50, 150, 310, 650 and 930 sec, and are shown in curves 3, 4, 5, 6, 7and 8, respectively. After reaching photosteady state, the sample was irradiated with UV light (curve 9) to produce all-*trans*-retinal bound form.

(B) Difference spectra calculated by subtracting curve 2 from curves 3-9 in (A). (C) The b-spectra of photoreactions calculated by SVD analysis and global fitting of V-spectra. The b-spectra were obtained by SVD analysis based on the difference spectra in (B) as described previously [6]. Only one kinetic component was extracted (-B1, 164.8 sec), which was consistent with the reaction induced by the UV light irradiation shown in Fig. 3B. The B0 spectrum, which is the spectrum extrapolated to infinite time, shows the photo-equilibrium state. Calculated residuals between B0 and curve 8 in (B) are shown in blue. (D) Calculated singular values obtained by SVD analysis.

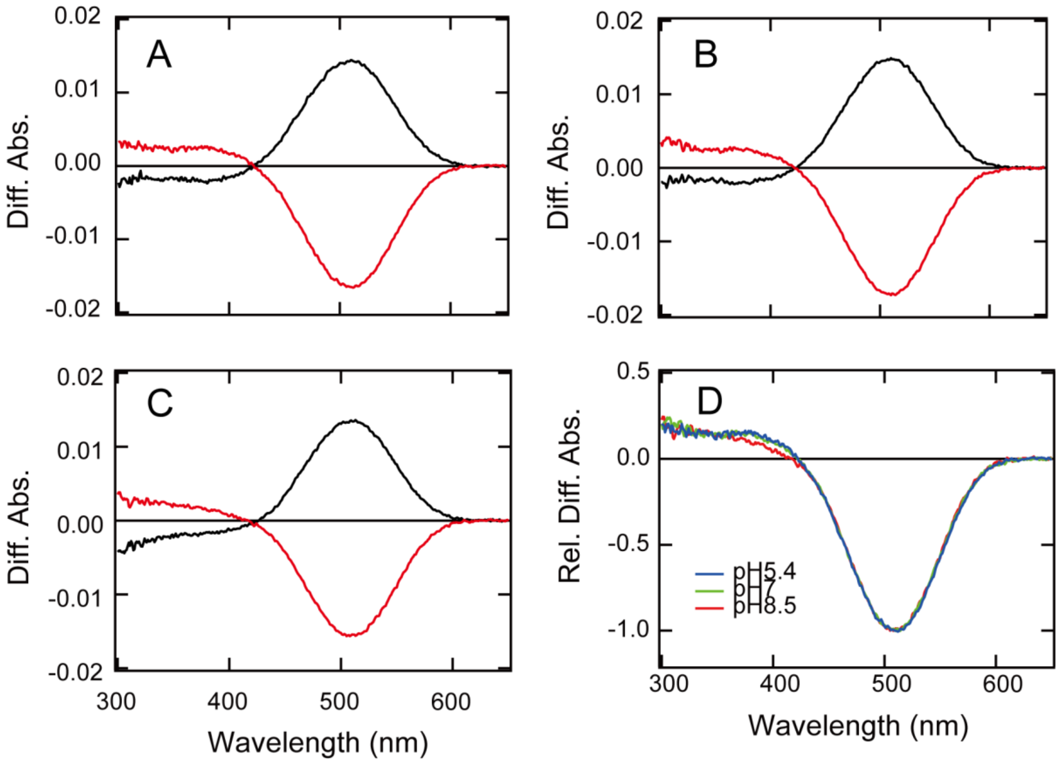

Fig. S6　pH dependent change of photoreactions of Rh7-Cap.

(A-C) Photoreactions of Rh7-Cap induced by UV light and subsequent yellow light irradiations at pH 5.4 (A), 7.0 (B) and 8.5 (C).　The black curves show difference spectra calculated by subtracting the spectra before irradiation from those after UV light irradiation.　The red curves show difference spectra calculated by subtracting the spectra after UV light irradiation from those after subsequent yellow light (>500 nm) irradiation.　(D) Superposition of difference spectra shown by red curves in (A)-(C).　Spectra were normalized to be ~1.0 at the negative maximum.

References

1 Katoh, K., Misawa, K., Kuma, K. & Miyata, T. MAFFT: a novel method for rapid multiple sequence alignment based on fast Fourier transform. *Nucleic Acids Res.* **30**, 3059-3066 (2002).

2 Stamatakis, A. RAxML version 8: a tool for phylogenetic analysis and post-analysis of large phylogenies. *Bioinformatics* **30**, 1312-1313, doi:10.1093/bioinformatics/btu033 (2014).

3 Whelan, S. & Goldman, N. A general empirical model of protein evolution derived from multiple protein families using a maximum-likelihood approach. *Mol. Biol. Evol.* **18**, 691-699 (2001).

4 Yang, Z. Maximum likelihood phylogenetic estimation from DNA sequences with variable rates over sites: approximate methods. *J. Mol. Evol.* **39**, 306-314 (1994).

5 Felsenstein, J. Confidence-limits on phylogenies - an approach using the bootstrap. *Evolution* **39**, 783-791, doi:Doi 10.2307/2408678 (1985).

6 Kojima, K., Imamoto, Y., Maeda, R., Yamashita, T. & Shichida, Y. Rod visual pigment optimizes active state to achieve efficient G protein activation as compared with cone visual pigments. *J. Biol. Chem.* **289**, 5061-5073, doi:10.1074/jbc.M113.508507 (2014).
